# Genome-wide analysis uncovers tomato leaf lncRNAs transcriptionally active upon *Pseudomonas syringae* pv. *tomato* challenge

**DOI:** 10.1038/s41598-021-04005-0

**Published:** 2021-12-31

**Authors:** Hernan G. Rosli, Emilia Sirvent, Florencia N. Bekier, Romina N. Ramos, Marina A. Pombo

**Affiliations:** 1 Instituto de Fisiología Vegetal, INFIVE, Universidad Nacional de La Plata, CONICET, La Plata, Buenos Aires Argentina; 2 Facultad de Ciencias Exactas, Universidad Nacional de La Plata, La Plata, Buenos Aires Argentina

**Keywords:** Plant sciences, Plant immunity

## Abstract

Plants rely on (in)direct detection of bacterial pathogens through plasma membrane-localized and intracellular receptor proteins. Surface pattern-recognition receptors (PRRs) participate in the detection of microbe-associated molecular patterns (MAMPs) and are required for the activation of pattern-triggered immunity (PTI). Pathogenic bacteria, such as *Pseudomonas syringae* pv. *tomato* (*Pst*) deploys ~ 30 effector proteins into the plant cell that contribute to pathogenicity. Resistant plants are capable of detecting the presence or activity of effectors and mount another response termed effector-triggered immunity (ETI). In order to investigate the involvement of tomato’s long non-coding RNAs (lncRNAs) in the immune response against *Pst*, we used RNA-seq data to predict and characterize those that are transcriptionally active in leaves challenged with a large set of treatments. Our prediction strategy was validated by sequence comparison with tomato lncRNAs described in previous works and by an alternative approach (RT-qPCR). Early PTI (30 min), late PTI (6 h) and ETI (6 h) differentially expressed (DE) lncRNAs were identified and used to perform a co-expression analysis including neighboring (± 100 kb) DE protein-coding genes. Some of the described networks could represent key regulatory mechanisms of photosynthesis, PRR abundance at the cell surface and mitigation of oxidative stress, associated to tomato-*Pst* pathosystem.

## Introduction

Plants are under the attack of different kind of pathogens and this provokes economical losses all around the world^1^. However, to defend themselves they possess a diversified innate immune system that consists in membrane and cytoplasmic receptors that are able to detect different pathogen features^2,3^. Pattern recognition receptors (PRRs) are on the surface of the plant cell and can recognize microbe-associated molecular patterns (MAMPs), activating an immune response named pattern-triggered immunity (PTI)^4^. This response includes production of reactive oxygen species (ROS), callose deposition into the apoplast, activation of MAP kinase cascades, increase of intracellular calcium concentration and transcriptional reprograming^5–8^.

Pathogenic bacteria such as *Pseudomonas syringae* pv. *tomato* (*Pst*) can introduce inside the plant cell cytoplasm effector proteins that are able to undermine PTI and also interfere with cellular processes for the promotion of their own growth, multiplication in the apoplast and virulence^9,10^. However, some plants have acquired resistance proteins (R proteins), most of them nucleotide-binding leucine-rich repeat proteins (NLRs) that can directly or indirectly detect some of these effectors. After this detection, they activate another immune response called effector-triggered immunity (ETI) or more recently named NLR-triggered immunity (NTI)^11,12^. ETI activation also includes ROS production, MAPK signaling, electrolyte leakage into the apoplast and transcriptional reprograming^13,14^, but it is characterized for the development of a hypersensitive response (HR) that culminates in programmed cell death (PCD)^15^. In addition, some effectors are involved in the suppression of this immune response^16^.

Tomato (*Solanum lycopersicum*) is an economically important crop that is produced all around the world. The interaction between tomato and *Pseudomonas syringae* pv. *tomato* (*Pst*), the causal agent of tomato speck disease, is used for the study of the molecular mechanisms implicated in bacterial pathogenesis and plant defenses^17,18^. Most of the transcriptional changes that occur upon *Pst*-mediated PTI activation in tomato are due to the perception of flagellin, the main component of bacterial flagella^7^. Tomato recognizes two epitopes of flagellin, flg22 and flgII-28, which are detected by the receptors FLS2 and FLS3, respectively^19–21^.

*Pst* DC3000 can introduce more than 30 effectors into the plant cell^22^. Two of them, AvrPto and AvrPtoB, interfere with PTI signaling right after MAMP detection^7,23^. However, resistant tomatoes can detect these two effectors through a protein kinase Pto that jointly with the NLR Prf, activate a strong ETI^18,24–26^.

The development of high sensitive sequencing technologies such as RNA-seq has allowed the identification of new transcripts, much of them not derived from annotated protein coding-genes^27^. For some time they were considered as “junk DNA”, but then more and more studies supported the idea that some of these non-coding RNAs (ncRNAs) possess important regulatory functions in different cellular processes^27^. Long non-coding RNAs (lncRNAs) are a subset of ncRNAs with an established size of 200 bp or more^28^. They are transcribed from diverse regions in the genome and according to this are classified in intergenic, intronic, overlapping with coding genes, sense and antisense, among others^29^.

Depending on their location inside the cell, they are believed to modulate different processes. Nuclear lncRNAs can regulate transcription of protein coding genes through chromatin modification, recruitment of transcription inhibitors or enhancers, enabling the proximity between enhancer sequences and transcription start sites and modulate alternative splicing by interacting with different splicing factors^30^. In the case of the cytoplasmic lncRNAs, they have been implicated in messenger RNA (mRNA) stability for example, acting as “sponges” of micro RNA (miRNA) avoiding their target mRNA degradation or producing small interference RNAs (siRNA) after being cut by a miRNA, that can subsequently lead to the degradation of other mRNAs^31^. Some lncRNAs interact with ribosomal proteins and therefore regulate mRNA translation to protein^32,33^.

Although less studied than in humans, lncRNAs are rising as important players in plants too^32,34,35^. In this sense, they have been involved in the regulation of different biological processes such as phosphorous nutrition deficiency^36^, sexual reproduction^37^, vernalization and floral timing^38,39^, abiotic^40^ and biotic stresses^41,42^.

Related with plant–pathogen interactions, previously and using microarrays, lncRNAs with higher expression after plant treatment with elf 18 (MAMP derived from the elongation factor Tu) were identified in Arabidopsis^41^. Then, ELF18-INDUCED LONG-NONCODING RNA1 (ELENA1) was functionally characterized as an intergenic lncRNA with active transcription after elf18 and flg22 perception. Plants with reduced levels of ELENA1 were more susceptible to *Pst* DC3000, while plants over-expressing ELENA1 developed an opposite phenotype, showing that this lncRNA acts as a positive regulator of plant defenses against this pathogen^42^.

Particularly in tomato, several lncRNAs were identified as expressed during fruit ripening^43,44^. In addition, tomato lncRNAs have been described as associated to interactions with virus^45,46^, viroid^47^ and the oomycetes *Phytophthora infestans*^48–51^. Until now, there are no reports of lncRNA with induced expression in the tomato-*Pst* pathosystem.

In the present work, we re-analyzed previously published RNA-seq data from tomato, derived from a large set of treatments/conditions^7,52,53^. Through this approach we were able to identify and characterize lncRNAs that are expressed in tomato leaves and determine those differentially expressed in early PTI (30 min), late PTI (6 h) and ETI at 6 h. By means of transcriptional co-regulation analysis including lncRNAs and protein-coding genes, a group of relevant networks were identified. Some of these could be part of the mechanisms behind the regulation of processes such as of photosynthesis, PRR abundance at the plasma membrane and oxidative stress response, upon *Pst* challenge in tomato plants.

## Results

### Tomato lncRNA identification

For our analysis we used a set of previously generated RNAseq data that includes 11 different treatments/controls (flg22, flgII-28 or different Pseudomonas spp. strains), each with three biological replicates (Table S1). The selection of these conditions was motivated by the fact that through certain comparisons we could capture lncRNA transcriptomic changes associated to early and late PTI (30 min and 6 h), ETI and the effect of two *Pst* DC3000 effectors (AvrPto and AvrPtoB) in suppressing PTI. We identified 22,595 novel tomato transcripts which were used as input for a pipeline (see “Materials and methods”) that allowed the prediction of 2609 putative lncRNAs transcriptionally active in tomato leaves under these conditions (Table S2). We investigated the degree of overlap between our predicted lncRNAs for the tomato-*Pst* pathosystem and those available from previous works using tomato under different conditions. Already identified tomato leaf lncRNAs included those detected upon challenge with Tomato yellow curl leaf virus (TYCLV)^45,46,54^ or *Phytophthora infestans*^48,55^, those available for tomato in CANTATA database^56^ and four predicted tomato *TRANS-ACTING SIRNA3* (*TAS3*) transcripts^57^. Performing a local blastn^58^ using our predicted lncRNAs as query and those derived from previous works as database, we found that 1247 (47.7%) of query sequences had at least one hit, with an overall average identity of 97.6% (Table S3). The remaining transcripts without match to those previously identified could account for lncRNAs that are transcriptionally active upon elicitation of tomato immune response by *Pseudomonas* spp. or MAMPs challenges and time-points used in this work (30 min and 6 h). It is worth mentioning that we cannot assume these “novel” lncRNAs we identified are specific of the bacterial pathosystem under study. We continued our analysis with all the lncRNAs predicted in this work, regardless of their being previously identified.

Analysis of the number of isoforms, indicated that the large majority of lncRNA genes (2141; 82%) encoded for a single isoform (Fig. S1). We defined a lncRNA as “expressed” if it had ≥ 3 FPKM (fragments per kilobase per million mapped reads) in at least one of the 11 conditions analyzed. From the 2609 lncRNAs we predicted with our pipeline, only these “expressed” lncRNAs (2048; Table S4) were considered from this point on, unless stated otherwise.

### Global characterization of tomato lncRNAs

**Figure 1:**
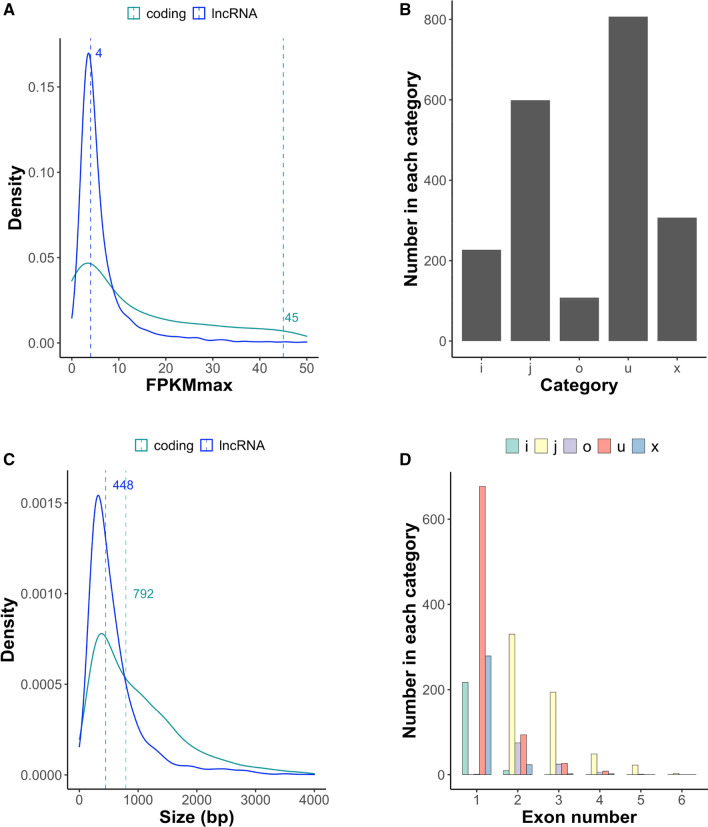
Global characterization of tomato lncRNAs and comparison with protein-coding genes. (**A**) Transcript abundance distribution considering maximum FPKM (FPKMmax) value from all samples and excluding those with FPKMmax = 0. The position of median value is indicated for each distribution. (**B**) Expressed lncRNAs (FPKM ≥ 3 in at least one sample) in categories based on their relationship to annotated tomato transcripts^59^. (**C**) Transcript size distribution of expressed lncRNAs and tomato protein-coding genes. The position of median value is indicated for each distribution. (**D**) Gene structure of expressed lncRNAs falling in the different categories based on their relationship to annotated tomato transcripts.

We analyzed the distribution of expression levels of protein-coding and lncRNA transcripts, excluding those with rounded average FPKM = 0 (Fig. 1A). LncRNAs’ FPKM median value was nearly tenfold smaller than that for protein-coding tomato genes, indicating that lncRNAs have overall lower expression levels as previously shown for Arabidopsis ^34^. From the 2048 expressed lncRNAs identified the most abundant categories, in terms of their relationship to annotated transcripts^59^, were *u* (unknown, intergenic transcript) and *j* (potentially novel isoform with at least one splice junction shared with reference transcript), while category *o* (generic exonic overlap with a reference transcript) was the least represented (Fig. 1B). We investigated how protein-coding and lncRNA transcript size distribution compared. Protein-coding transcripts’ distribution shifted towards larger sizes with a median value that almost doubled the observed for lncRNAs (Fig. 1C).

To further characterize the identified tomato lncRNAs we studied the number of exons for transcripts in each category (Fig. 1D). For categories *i*, *u* and *x* most transcripts contained a single exon while for those in categories *j* and *o*, 2-exon transcripts were most abundant. Overall, 1-exon transcripts accounted for 1174 lncRNAs (57% of all expressed lncRNAs).

**Figure 2:**
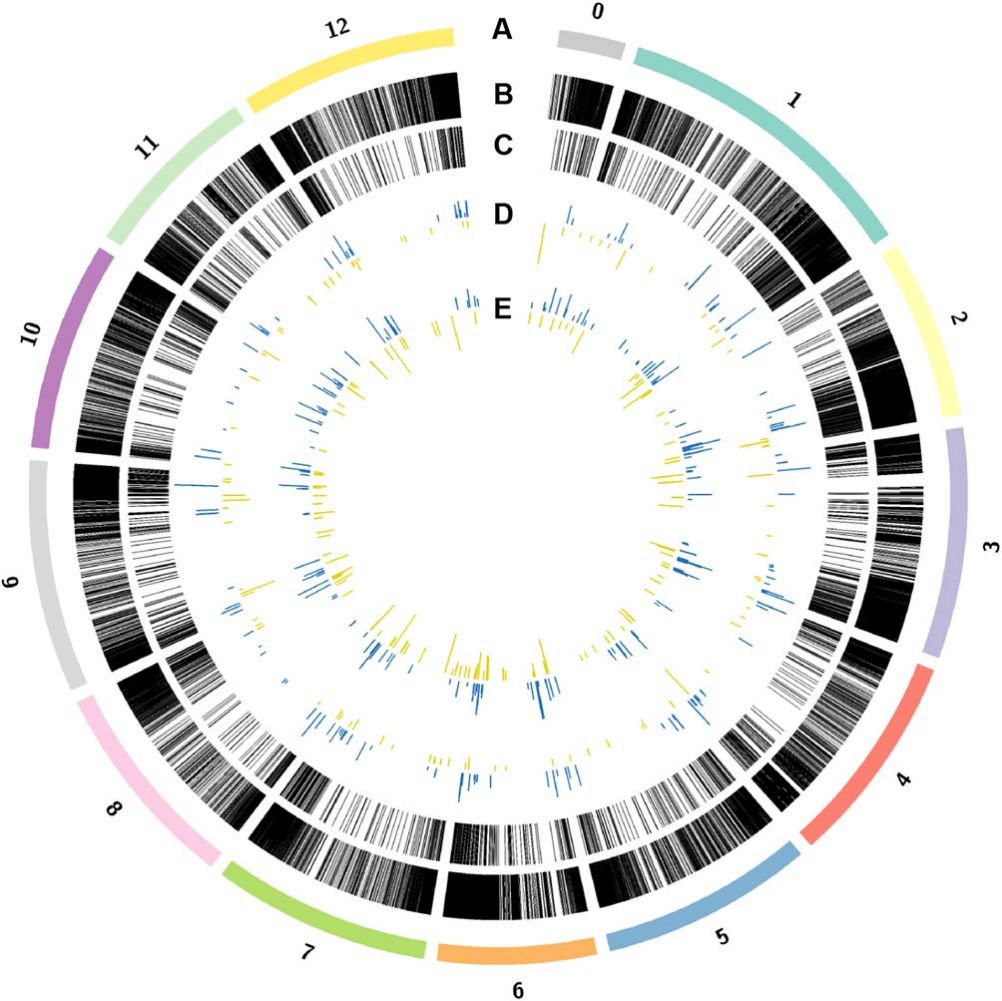
Genomic distribution of lncRNAs and protein-coding genes. (**A**) tomato chromosomes, (**B**) protein-coding genes, (**C**) lncRNAs, (**D**) DELs mock_flgII-28_6h vs flgII-28_6h (PTI-flgII28, 6 h), (**E**) DELs for *Pst* DC3000 Δ*fliC*Δ*avrPto*Δ*avrPtoB* vs *Pst* DC3000 Δ*fliC* in PtoR background (ETI, 6 h). Induced and suppressed transcripts are indicated with blue and yellow lines, respectively. The length of these lines is proportional to the transcript fold-change in each comparison.

The distribution of lncRNAs in tomato chromosomes did not differ from the one observed for protein-coding genes (Fig. S2), ranging from 7 to 12% for chromosomes 1–12. We then analyzed the position within each chromosome where protein-coding (Fig. 2, lane B) and lncRNAs (Fig. 2, lane C) reside. Both types of transcripts are encoded mainly at the beginning and ending of each chromosome. These findings indicate there are no obvious lncRNA-specific hot spots in the genome and that these are encoded in the same regions as protein-coding genes.

### LncRNAs’ expression changes associated to tomato immunity

**Figure 3:**
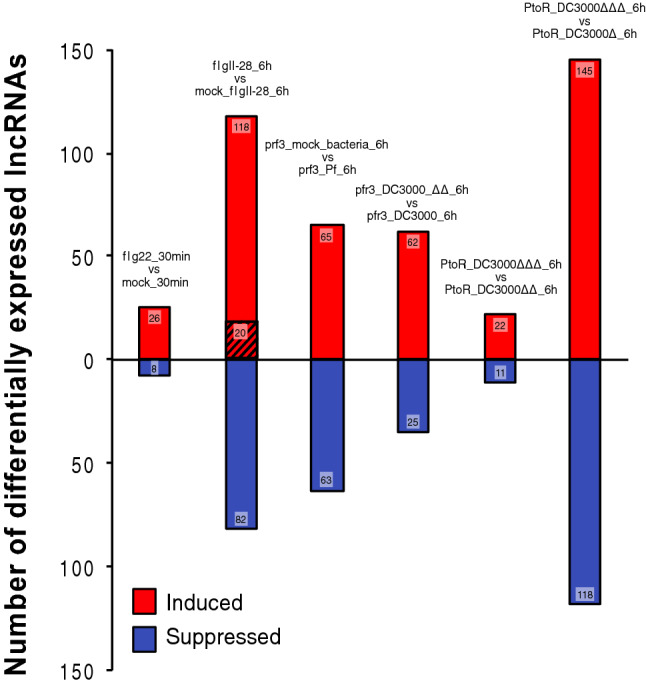
Differentially expressed lncRNAs (DELs). For each comparison, number of induced and suppressed DELs (q-value < 0.05 and |log2 fold-change| ≥ 1) is shown inside the graph bar. Striped pattern in flgII-28_6h vs mock_flgII-28_6h represents number of FIRE DELs. prf3_Pf_6h (*Pseudomonas fluorescens* in RG-*prf3* background); prf3_DC3000_6h (*Pst* DC3000 in RG-*prf3* background); prf3_DC3000ΔΔ_6h (*Pst* DC3000 Δ*avrPto*Δ*avrPtoB* in RG-*prf3* background); PtoR_DC3000Δ_6h (*Pst* DC3000 Δ*fliC* in RG-PtoR background); PtoR_DC3000ΔΔ_6h (*Pst* DC3000 Δ*avrPto*Δ*avrPtoB* in RG-PtoR background); PtoR_DC3000ΔΔΔ_6h (*Pst* DC3000 Δ*fliC*Δ*avrPto*Δ*avrPtoB* in RG-PtoR background).

Setting a cut-off of q-value < 0.05 and |log2 fold-change| ≥ 1, we established differentially expressed lncRNAs (DELs) for the comparisons of interest (Table S4 and Fig. 3). Early flg22-associated PTI induction lead to a small set of DELs (26 up- and 8 down-regulated). Contrastingly, for flgII-28 challenge at 6 h time-point the number of DELs was clearly larger, suggesting a stronger immune response at the transcriptional level (118 up- and 82 down-regulated). Leaf infiltration with the strong PTI inducer, *Pseudomonas fluorescens* 55, lead to a number of DELs that were fewer than those identified for flgII-28. This finding is in agreement with the data that derives from the same treatments, but for protein-coding transcripts differentially expressed genes (DEGs)^7^. We compared DC3000 vs DC3000 Δ*avrPto*Δ*avrPtoB* in RG-*prf3* susceptible plants. With this comparison, which accounts for the effect of AvrPto and AvrPtoB effectors at the transcriptional level, we observed that up-regulation of lncRNAs prevailed over down-regulation. This same trend had been previously found for protein-coding genes^7^.

A group of transcripts of particular interest are those induced by flgII-28 treatment and suppressed by AvrPto and/or AvrPtoB effectors (DC3000 < DC3000 Δ*avrPto*Δ*avrPtoB* in RG-*prf3* susceptible plants). This group of transcripts were previously termed FIRE (flagellin-induced, repressed by effectors) which allowed the identification of a tomato wall associated kinase, SlWak1, that participates in the immunity against *Pst*^7,60^. We were able to identify a set of 20 FIRE lncRNAs (Table S4 and Fig. 3) which accounts for ~ 57% of those suppressed by AvrPto and/or AvrPtoB in RG-*prf3*. In contrast, in the case of protein-coding genes, this percentage is considerably higher (~ 91%)^7^.

AvrPto- and/or AvrPtoB-induced ETI (DC3000 Δ*fliC*Δ*avrPto*Δ*avrPtoB* vs. DC3000 Δ*fliC* in PtoR background) lead to the highest numbers of DELs of all treatments analyzed, both up- and down-regulated, while flagellin-associated PTI was associated to a milder transcriptional response (Table S4 and Fig. 3). A similar behavior was observed for protein-coding genes under the same challenges^52^.

**Figure 4:**
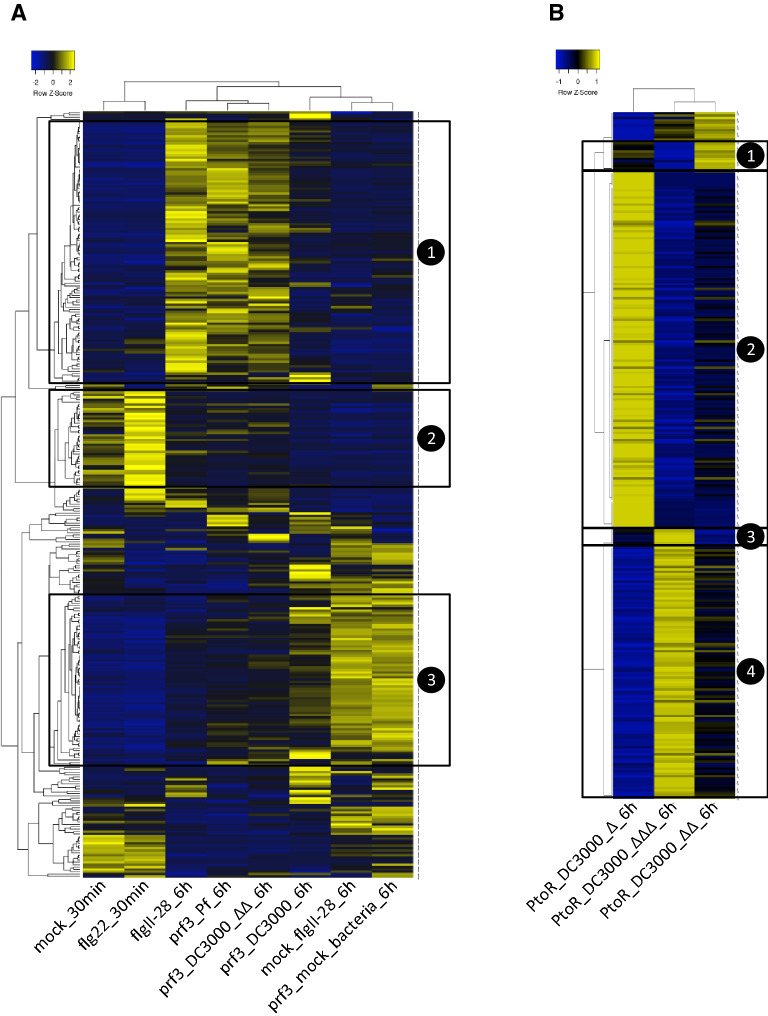
Clustering analysis based on lncRNAs and treatments. LncRNAs included in each cluster had at least one comparison of interest, indicated in Fig. 3, with q-value < 0.05. (**A**) Cluster for treatments in RG-*prf3* plants with the following groups highlighted: 1, induced by PTI at 6 h; 2, induced by flg22 at 30 min; 3, suppressed by PTI at 6 h. (**B**) Cluster for treatments in RG-PtoR plants with the following groups highlighted: 1, strong PTI induction; 2 strong ETI induction; 3, strong PTI suppression; 4, strong ETI suppression. *Pst* DC3000 mutants’ nomenclature is described in Fig. 3 legend.

In order to globally analyze lncRNA transcriptional changes, we performed separate clustering analysis data deriving from RG-*prf3* and RG-PtoR tomato lines’ challenges. Treatments in RG-*prf3* plants formed three clear clusters that can be categorized as 30 min time-points, PTI-inducing treatments and mock treatments; these last two, at 6 hai (Fig. 4A). It is worth mentioning that grouping along with mock treatments, was DC3000 challenge in RG-*prf3* plants, which can be associated to the effect of AvrPto and/or AvrPtoB in suppressing PTI response at the transcriptional level of protein coding genes^7^. Distinct transcript clusters included: 1, PTI induced at 6 hai (including some FIRE lncRNAs); 2, early flg22 induced; 3, PTI suppressed at 6 hai (Fig. 4A). Within cluster 1, some transcripts following FIRE transcriptional changes can be visualized. In the case of treatments in RG-PtoR background clustering (PtoR_DC3000ΔΔΔ grouping with PtoR_DC3000ΔΔ) was in agreement with having found a larger number of DELs for the comparison PtoR_DC3000ΔΔΔ vs PtoR_DC3000Δ, than in PtoR_DC3000ΔΔΔ vs PtoR_DC3000ΔΔ (Figs. 3 and 4B). Transcript clustering allowed the identification of groups of lncRNAs associated to strong PTI/ETI suppression/induction (Fig. 4B).

### Evaluation of lncRNAs’ expression by RT-qPCR

**Figure 5:**
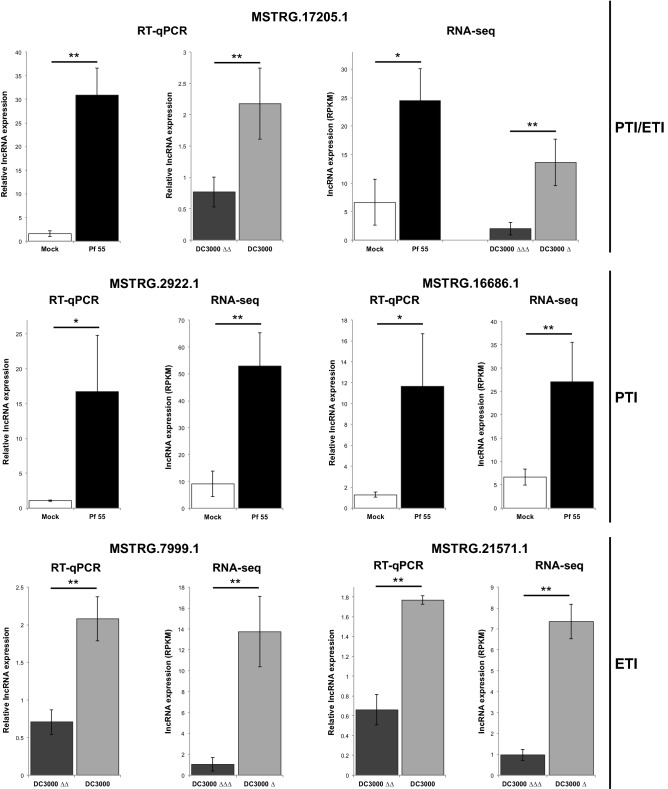
Expression analysis of selected DELs by RT-qPCR. Analyzed transcripts include PTI-, ETI- and PTI/ETI-responsive lncRNAs. Bars represent the median of 3 biological replicates and 3 technical replicates each, with their corresponding standard deviation. For RT-qPCR results, * or ** represent statistical differences using Student's *t*-test 0.05 and 0.01, respectively. For RNA-seq results, * represents q-value < 0.05 and ≥ twofold change, while ** q-value < 0.01 and |log2 fold-change| ≥ 1. Pf 55 (*Pseudomonas fluorescens* 55); PtoR_DC3000Δ_6h (*Pst* DC3000 Δ*fliC* in RG-PtoR background); PtoR_DC3000ΔΔΔ_6h (*Pst* DC3000 Δ*fliC*Δ*avrPto*Δ*avrPtoB* in RG-PtoR background).

For this purpose we selected several lncRNAs: two induced by *P. fluorescens* 55 treatment (PTI, prf3_mock_bacteria_6h vs prf3_Pf_6h), two induced by ETI (DC3000 Δ*fliC*Δ*avrPto*Δ*avrPtoB* vs. DC3000 Δ*fliC* in PtoR background) and one induced by both immune responses. We challenged an independent set of RG-PtoR plants to induce PTI (mock vs *P. fluorescens* 55) and ETI (DC3000 Δ*avrPto*Δ*avrPtoB* vs DC3000) and sampled at the same time-point as the one used for the RNA-seq experiment (6 hai). For all selected lncRNAs we were able to detect their corresponding transcripts and to confirm their transcriptional changes upon PTI and/or ETI activation (Fig. 5).

### Gene ontology (GO) term analysis and co-expression networks

Transcriptional co-regulation of lncRNAs and neighboring protein-coding genes could help identify networks that are modulated by lncRNAs. Such regulation represents one of lncRNAs’ mechanisms to control gene expression (cis-action)^31^. In our case we were interested in identifying lncRNAs that modulate key protein-coding genes involved in tomato defense response against *Pst*. For up-regulated DELs identified in the comparisons mock_flgII-28_6h vs flgII-28_6h (PTI-flgII-28) and DC3000 Δ*fliC*Δ*avrPto*Δ*avrPtoB* vs. DC3000 Δ*fliC* in PtoR background (ETI-AvrPto/AvrPtoB), we identified those neighboring (within a 100 Kb genome region^48,61–63^) protein-coding genes whose transcriptional behavior was the same (up-regulated, positive co-regulation) or opposite (down-regulated, negative co-regulation) for these same comparisons, using |log2 fold-change| ≥ 1 and q-value < 0.05 as cut-offs. This set of genes was termed neighboring protein-coding co-regulated genes (NCG). Though gene ontology (GO) term analysis of PTI-flgII-28 induced DELs’ NCG with positive co-regulation (121 coding genes) resulted in no enrichment, “kinase activity” was one terms with the lowest p-value and was assigned to 10 NCGs (Table S5). Negatively co-regulated NCGs (82 total coding genes) were enriched in the term “photosynthesis” (Table S5), indicating that the corresponding DELs could be controlling transcript abundance of these NCGs and consequently leading to a suppression of the photosynthesis-related genes during PTI activation.

The analysis of the 221 NCGs with positive co-regulation with ETI up-regulated DELs, revealed that “transcription factor activity, sequence-specific DNA binding” was one of the terms with lowest p-value (Table S6). Again for negatively co-regulated NCGs we found an enrichment of “photosynthesis” term. Indicating that lncRNAs may participate in the negative modulation of photosynthesis-related genes during both PTI and ETI induction.

**Figure 6:**
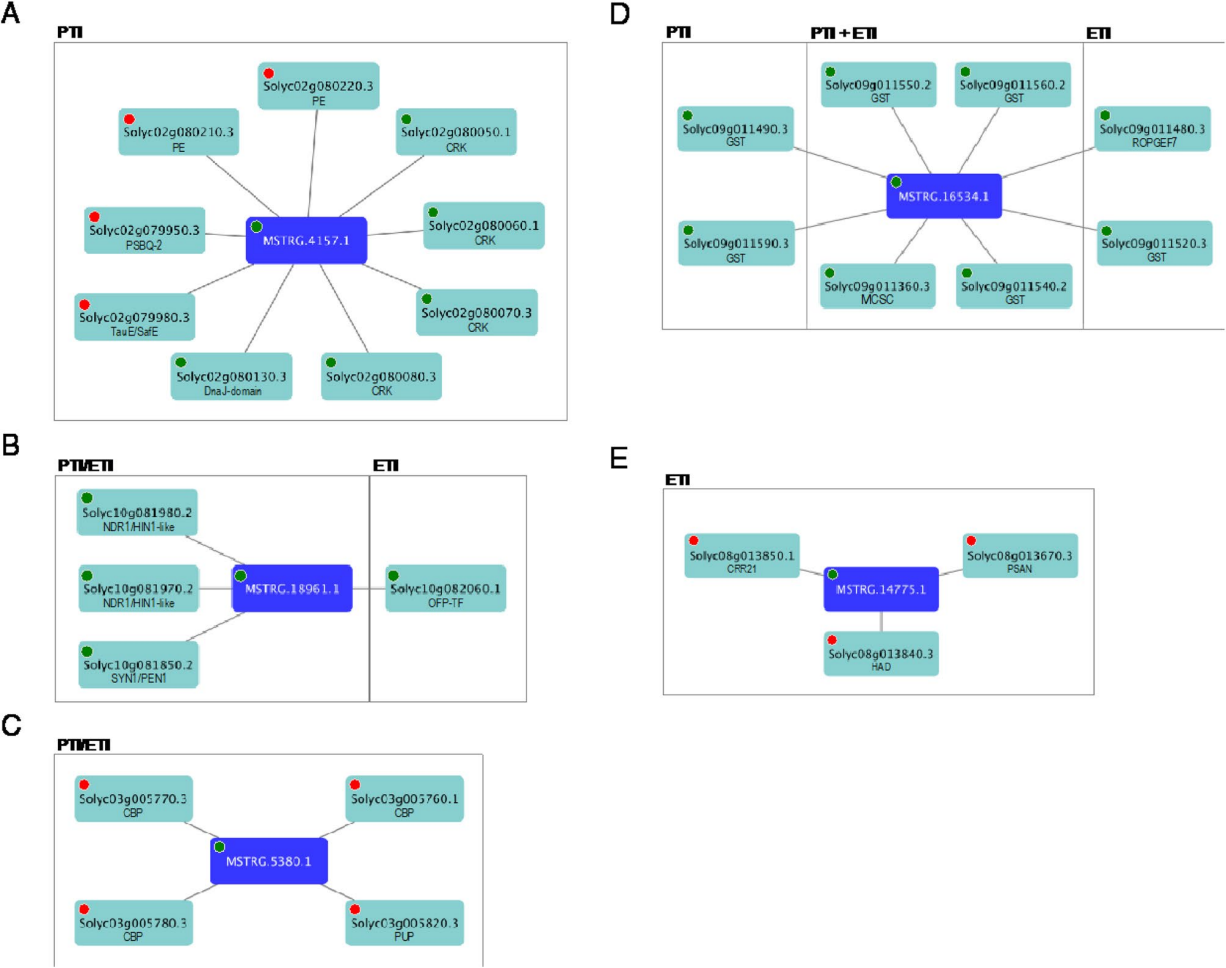
Selected DELs—neighboring protein-coding co-regulated genes (NCG) networks. For DELs found in the comparisons prf3_mock_flgII28_6h vs prf3_flgII-28_6h (PTI) and DC3000 Δ*fliC*Δ*avrPto*Δ*avrPtoB* vs DC3000 Δ*fliC* at 6 h in PtoR plants (ETI) their NCG were identified and used to generate the networks. (**A**) PSBQ-2 (Photosystem II subunit Q-2); TauE/SafE (Sulfite exporter TauE/SafE); PE (Pectinesterase); CRK (Cystein-rich receptor-like kinase); DnaJ-domain (Chaperone DnaJ-domain superfamily). (**B**) SYN/PEN1 (Syntaxin 1/Penetration 1); NDR1/HIN1-like (Arabidopsis non-race specific disease resistance gene 1/Harpin-induced gene 1); OFP-TF (Ovate family protein (OFP) transcription factor). (**C**) CBP (Chlorophyll a/b-binding protein); PUP (Purine permease). (**D**) GST (Glutathione S-transferase); ROPGEF7 (ROP (Rho of plants) guanine nucleotide exchange factor 7); MCSC (Mitochondrial substrate carrier family protein). (**E**) CRR21 (Chlororespiratory reduction 21); HAD (Haloacid dehalogenase-like hydrolase); PSAN (Photosystem I reaction center subunit PSI-N). Red and green circles indicate suppression or induction in the comparisons evaluated, respectively.

To further understand the relationships between lncRNAs and their co-regulated coding genes we performed a network analysis including PTI-flgII-28- and ETI-AvrPto/AvrPtoB-induced NCGs. Complete networks can be found in Figs. S3–S6, while selected ones are shown in Fig. 6. We could identify networks that are exclusive of PTI-flgII-28 (Fig. 6A) or ETI-AvrPto/AvrPtoB (Fig. 6E), but also common ones (Fig. 6B–D). MSTRG.4157.1, a category x and PTI-flgII-28-induced lncRNA, was member of one of the largest networks found. In this case NCGs included transcripts up- and down-regulated by PTI-flgII-28 activation. Four up-regulated NCGs encode for cystein-rich receptor-like kinases and one for a chaperone, while down-regulated NCGs included transcripts coding for cell wall degrading enzymes, a photosystem II subunit and a sulfite transporter (Fig. 6A).

The network of MSTG.18961.1 (Fig. 6B), an intronic lncRNA of Solyc10g081980.2, contained Solyc10g081850.2 whose closest protein in *Arabidopsis thaliana* is AT3G11820 (Penetration 1, PEN1) that has been recently proposed to have role in the accumulation of the receptor FLS2 at the plasma membrane^64^. Two Arabidopsis non-race specific disease resistance gene 1/Harpin-induced gene 1 (NDR1/HIN1)-like transcripts also belong to this network. Particularly interesting is Solyc10g081980.1 whose closest protein in A. thaliana is AT5G06320 (NDR1/HIN1-like 3, NHL3), a membrane-localized protein that when overexpressed leads to plants with increased resistance to pathogenic *Pst* DC3000^65^.

Three PTI-flgII-28- and ETI-AvrPto/AvrPtoB-suppressed NCGs coding for chlorophyll a/b-binding proteins (CBP) form a network with MSTRG.5380.1, a DEL induced by these same immune responses (Fig. 6C). In this network we also identified a transcript encoding for a purine permease. Another lncRNA, but only induced by ETI-AvrPto/AvrPtoB (Fig. 6E), shares a network with a transcript coding for CRR21 (Chlororespiratory reduction 21) which plays a role in chloroplast RNA editing of a subunit of the NAD(P)H complex, which is key for its function^66^. In addition, a transcript coding for PSAN (Photosystem I reaction center subunit PSI-N) was found to be part of this network. These lncRNAs could negatively impact the abundance of transcripts coding for key photosynthesis-related proteins.

Six transcripts coding for glutathione S-transferases (GST) that are induced by PTI-flgII-28- and/or ETI-AvrPto/AvrPtoB, belong to the network of MSTRG.16534.1, an intergenic lncRNA that may modulate these coding genes’ transcript abundance (Fig. 6D). GSTs have been shown to participate in plant immunity against different types of pathogens^67^.

## Discussion

Taking advantage of a large set of RNA-seq data we were able to identify lncRNAs transcriptionally active in tomato leaves challenged with MAMPs and bacterial strains. Input RNA-seq data for prediction of novel transcripts represented 25× coverage of tomato genome and a stringent pipeline was used for lncRNA identification. The reliability of our approach was confirmed by comparing our set of lncRNAs with others described for tomato in previous publications and by analyzing gene expression by another methodology (RT-qPCR) the predicted transcriptional changes associated to different bacterial challenges.

Differential gene expression analysis of lncRNAs revealed that challenges with fewest and largest number of up-/down-regulated transcripts were mostly in agreement with what was observed for protein-coding genes^7,52^. This analysis allowed the identification of FIRE lncRNAs, which represent a promising set of candidates for studying their involvement in tomato immunity against *Pst*.

Effectors AvrPto and AvrPtoB have the capacity to suppress early recognition stages of PTI^68^. In agreement with this most of the genes suppressed by these effectors (DC3000 < DC3000 Δ*avrPto*Δ*avrPtoB* in RG-*prf3* plants) should also be induced by PTI (mock < flgII-28). That is the case for protein-coding genes, with a 91% of genes suppressed by AvrPto/AvrPtoB that are also induced by flgII-28^7^. This percentage is clearly lower (57%) for the case of lncRNAs analyzed in this work using the same exact challenges. This means that there is a larger proportion of lncRNAs that are suppressed by these effectors that are not modulated by PTI activation. This may be evidence of virulence exerted by AvrPto and AvrPtoB through manipulation of key lncRNAs’ abundance, independently of their effect on PTI suppression.

To identify putative lncRNAs that may participate in plant immunity activation by modulating transcript abundance of neighboring protein-coding genes^30^, we performed a network analysis that included PTI-flgII-28- and ETI-AvrPto/AvrPtoB-induced NCGs. This analysis revealed several interesting groups of transcripts whose abundance could be modulated by lncRNAs. Nuclear encoded chloroplast-targeted genes (NECGs) have been shown to be down-regulated upon activation of PTI, ETI or challenge with a pathogenic bacterial strain^7,69,70^, though a reduction in photosynthetic activity is only observed in the last two situations^71^. We found at least one network with photosynthesis-related coding genes suppressed by ETI and not by PTI that could contribute to the differences observed in the status of chloroplast physiology between these immune responses.

Control of the abundance of membrane-localized of FLS2 receptor is key for modulating the perception of flg22. Several components of this control system have been identified^72–74^, including degradation of FLS2 through selective autophagy, mediated by ATG8 and orosomucoid proteins^75^. Recently, subunits EXO70B1/2 of exocyst complex have been shown to modulate trafficking of FLS2 to the plasma membrane and PEN1 may independently participate in this process^64^. We identified a PTI- and ETI-induced lncRNA whose NCGs included PEN1. It is possible that this lncRNA modulates the abundance of PEN1 transcript and consequently affects the levels of FLS2 at the plasma membrane. This up-regulation of a typically PTI-associated gene upon PTI and ETI induction is consistent with fairly recent findings that indicate there is a crosstalk between these two responses^76,77^.

Members of the GST protein family have been found to be transcriptionally induced upon PTI and ETI activation and contribute to mitigating oxidative stress^67^. We identified a set of 6 GSTs encoded in chromosome 9 of tomato, induced by PTI and/or ETI, that could potentially be transcriptionally regulated by MSTRG.16534.1. Tomato glutarredoxin *SlGRX*, which also contributes to preventing oxidative damage and promote resistance to *Phytophthora infestans*, can be induced by the neighboring lncRNA16397^48^. Further exploration of MSTRG.16534.1 network may shed light on a similar lncRNA-based control of oxidative damage.

To our knowledge our work represents the first report on tomato lncRNAs’ participation against a bacterial pathogen, such as *Pst*. We believe the generated information will contribute to finding key regulatory modules controlling important processes during plant-pathogen interactions.

## Methods

### Tomato leaf transcript prediction and quantification

Raw RNA-seq reads from 33 samples of tomato leaves challenged with flg22, flgII-28 or different *Pseudomonas* spp. strains detailed in Table S1 were retrieved from Sequence Read Archive (SRA; https://www.ncbi.nlm.nih.gov/sra) available at NCBI. The complete set used accounted for 475 M reads (~ 21 Gb; 25×, 828 Mb genome). Reads were aligned to tomato rRNA sequences retrieved from SILVA database^78^ using Bowtie^79^ (v1.2.2) with the option-v 3 to allow a thorough removal of rRNA contamination. Clean reads were mapped to the tomato genome (assembly 3.00)^80^ with Hisat2 program^81^ (v2.1.0). Transcript assemblies and quantification were performed using Stringtie^81^ (v1.3.3). Each alignment file was used to generate individual transcript assemblies, with the default setting of minimal transcript length of 200 bp, that were then merged into a single assembly by setting the option –merge. This merged assembly was used to estimate transcript abundance for each sample. Cuffcompare^82^ (v2.2.1) along with tomato gene models (ITAG3.2)^80^ allowed classifying 21,771 novel transcripts in class codes based on their relationship to annotated transcripts in the following categories of interest: *j*, potentially novel isoform (at least one splice junction shared with reference transcript); *i*, transcript falling entirely within a reference intron; *o*, generic exonic overlap with a reference transcript; *u*, unknown, intergenic transcript; *x*, exonic overlap with reference transcript on the opposite strand^59^. Differentially expressed transcripts were identified with DESeq2 software^83^ (v1.26.0) using raw count data.

### LncRNA identification and global characterization

Novel transcripts falling in the categories mentioned above were used as input for getorf stand-alone tool from EMBOSS (v6.6.0.0) which allowed the identification of 4,397 that had no open reading frame larger than 300 nt. From these, 2,677 had no homology to any peptide present in Pfam database (v31.0)^84^ using blastx (Expect-value > 1e − 3). We employed CPC2 tool^85^ to identify 2,668 transcripts with low coding potential. Finally to further remove transcripts that would not qualify as lncRNAs, we used batch sequence search tool from Rfam database^86^ to filter other types of genomic and plastidial RNAs. After this stringent pipeline we kept 2,609 transcripts as putative tomato lncRNAs.

We used local blastn (-evalue 1e − 10 -soft_masking ‘false’ -num_alignments 1) to compare our predicted lncRNA with those available from previous works in tomato: leaves challenged with *Tomato yellow curl leaf virus* (TYCLV)^45,46,54^ or *Phytophthora infestans*^48,55^; fruit pericarp tissue, roots infected with *Meloidogyne incognita* and leaves inoculated with *Potato spindle tuber viroid* from CANTATA database^56^; and four predicted tomato *TRANS-ACTING SIRNA3* (*TAS3*) transcripts^57^.

To generate a graphical representation of the genomic distribution of protein-coding and lncRNAs we used software package Circos^87^ (v0.69-8).

### Neighboring co-regulated genes’ identification, network generation and GO term analysis

Considering lncRNAs may modulate the expression of genes within a 100 kb up/down-stream region we identified their corresponding neighboring coding genes region^48,61–63^. Then for each DEL found in the comparisons mock_flgII-28_6h vs flgII-28_6h (PTI-flgII-28) and DC3000 Δ*fliC*Δ*avrPto*Δ*avrPtoB* vs DC3000 Δ*fliC* at 6 h in PtoR plants (ETI-AvrPto/AvrPtoB) we identified those neighboring protein-coding co-regulated genes (NCG), defined as having the same or opposite trend (|log2 fold-change| ≥ 1, q-value < 0.05). This information was used to generate NCG networks with Cytoscape program^88^ (v3.8.2). We subjected the lists of NCGs identified for PTI-flgII-28 and ETI-AvrPto/AvrPtoB comparisons, to a GO term analysis using AgriGO v2.0^89^ with default settings, ITAG3.2 as background and Plant GO Slim as gene ontology type. We analyzed separately those NCGs positively and negatively co-regulated in each comparison.

### Clustering analysis

Given that the RNA-seq data used in this work derives from two separate experiments using different Rio Grande tomato backgrounds (RG-PtoR and RG-*prf3*, see Table S1), we performed two independent clustering analyses for each of them with Heatmapper online tool^90^, using average linkage (clustering method) and Spearman rank correlation (distance measurement method). Input data in both cases were FPKM values of those expressed lncRNA (≥ 3 FPKM in at least one condition) with at least one q-value < 0.05 in any of the comparisons of interest.

### Bacterial challenge and RT-qPCR assay

Four-week old Rio Grande PtoR (RG-PtoR) tomato plants, kindly provided by Prof. Gregory B. Martin, were syringe infiltrated on leaflets of their third true leaves, with mock solution (10 mM MgCl_2_) or the following suspensions: 10^8^ colony forming units (cfu)/mL of *Pseudomonas fluorescens* 55 (Pf 55), 5 × 10^6^ cfu/mL *Pst* DC3000 (DC3000) and 5 × 10^6^ cfu/mL *Pst* DC3000 Δ*avrPto*Δ*avrPtoB* (DC3000 ΔΔ)*.* Mock vs Pf 55 accounts for PTI induction, while DC3000 ΔΔ vs DC3000 for AvrPto/AvrPtoB ETI induction. Six hours post infiltration (hpi) leaf tissue was frozen with N_2_(l) and stored at -80 °C until use. Total RNA isolation and cDNA synthesis were performed as described previously^91^. RT-qPCR reaction mix consisted of: 5 μL of 2× SYBR Green/ROX Master Mix (PB-L, Bio-Logic Products), 2 μL of 2 μM primer mix, 0.2 μL of 50× ROX, 2 μL of a diluted 1:10 cDNA and miliQ H_2_O to complete 10 μL final volume. Cycling conditions were 94 °C for 5 min and 45 cycles of 92 °C for 15 s, 60 °C for 20 s and 72 °C for 15 s. For gene expression analysis we selected one PTI/ETI-induced (MSTRG.17205.1), two PTI-induced (MSTRG.2922.1 and MSTRG.16686.1) and two ETI-induced (MSTRG.7999.1 and MSTRG.21751.1). Two reference genes (*ARD2* and *VIN3*) were used for normalization^53^. A list of primers used in this work can be found in Table S7. This study complied with local and national regulations.

## Supplementary Information

Supplementary Figures.

Supplementary Table S1.

Supplementary Table S2.

Supplementary Table S3.

Supplementary Table S4.

Supplementary Table S5.

Supplementary Table S6.

Supplementary Table S7.
