# Supplementary material for: Genome-wide analysis uncovers tomato leaf lncRNAs transcriptionally active upon Pseudomonas syringae pv. tomato challenge
Source: Sci Rep. 2021 Dec 31;11:24523. doi: 10.1038/s41598-021-04005-0 (PMC8720101; doi:10.1038/s41598-021-04005-0)
Supplement: Supplementary file 1 — Supplementary Figures. [file 41598_2021_4005_MOESM1_ESM.pptx]

## Slide 1
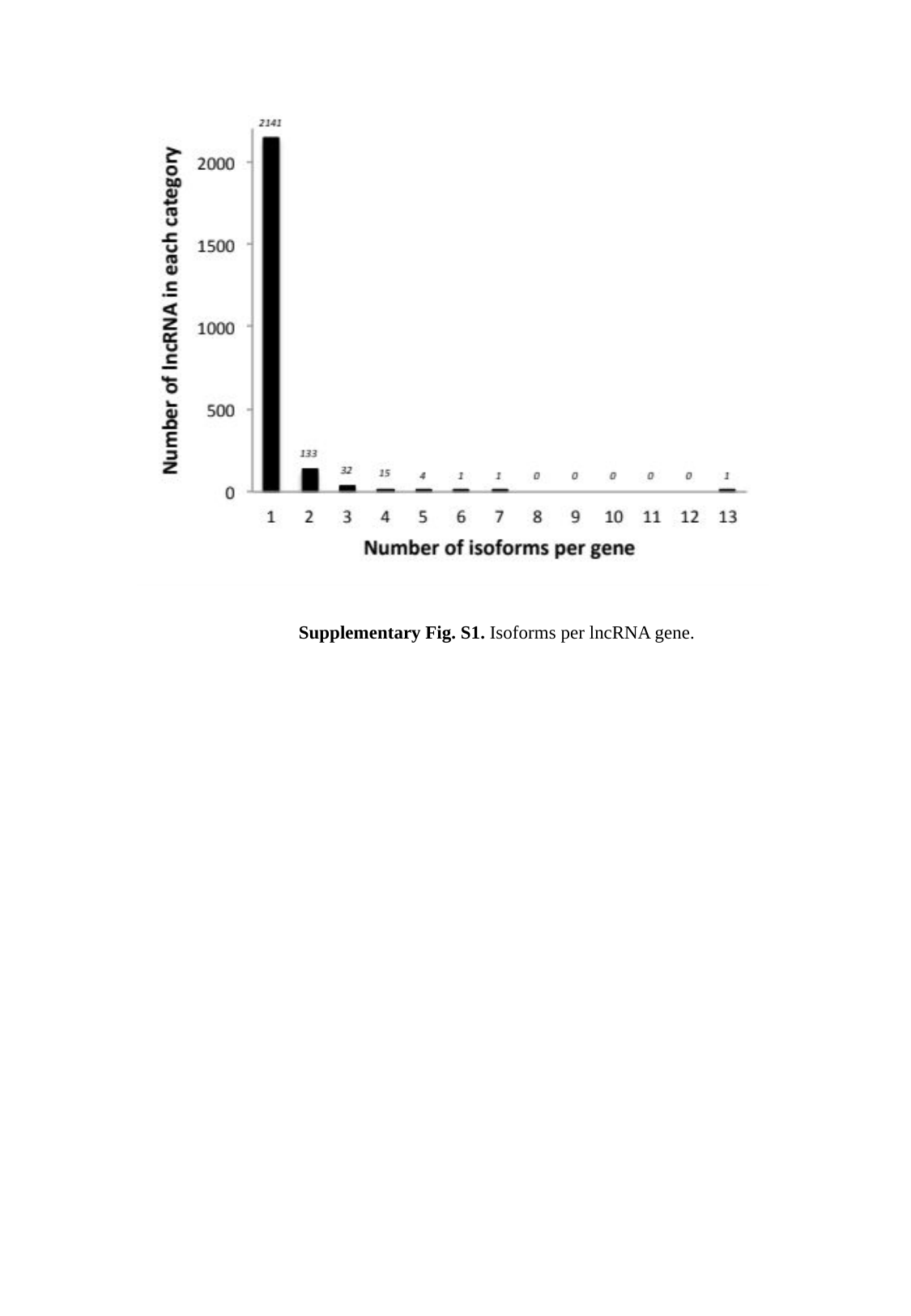

Supplementary Fig. S1. Isoforms per lncRNA gene.

## Slide 2
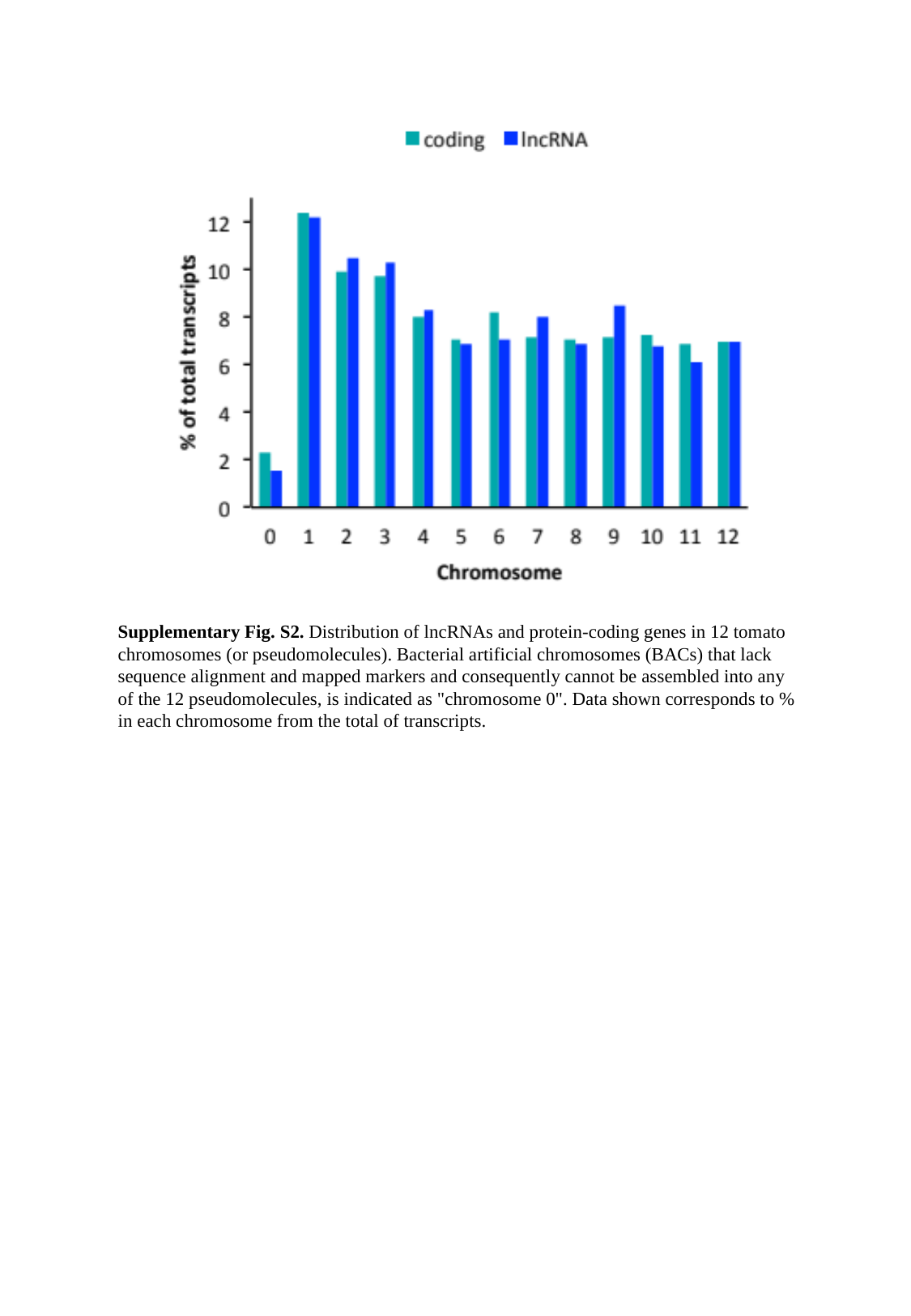

Supplementary Fig. S2. Distribution of lncRNAs and protein-coding genes in 12 tomato chromosomes (or pseudomolecules). Bacterial artificial chromosomes (BACs) that lack sequence alignment and mapped markers and consequently cannot be assembled into any of the 12 pseudomolecules, is indicated as "chromosome 0". Data shown corresponds to % in each chromosome from the total of transcripts.

## Slide 3
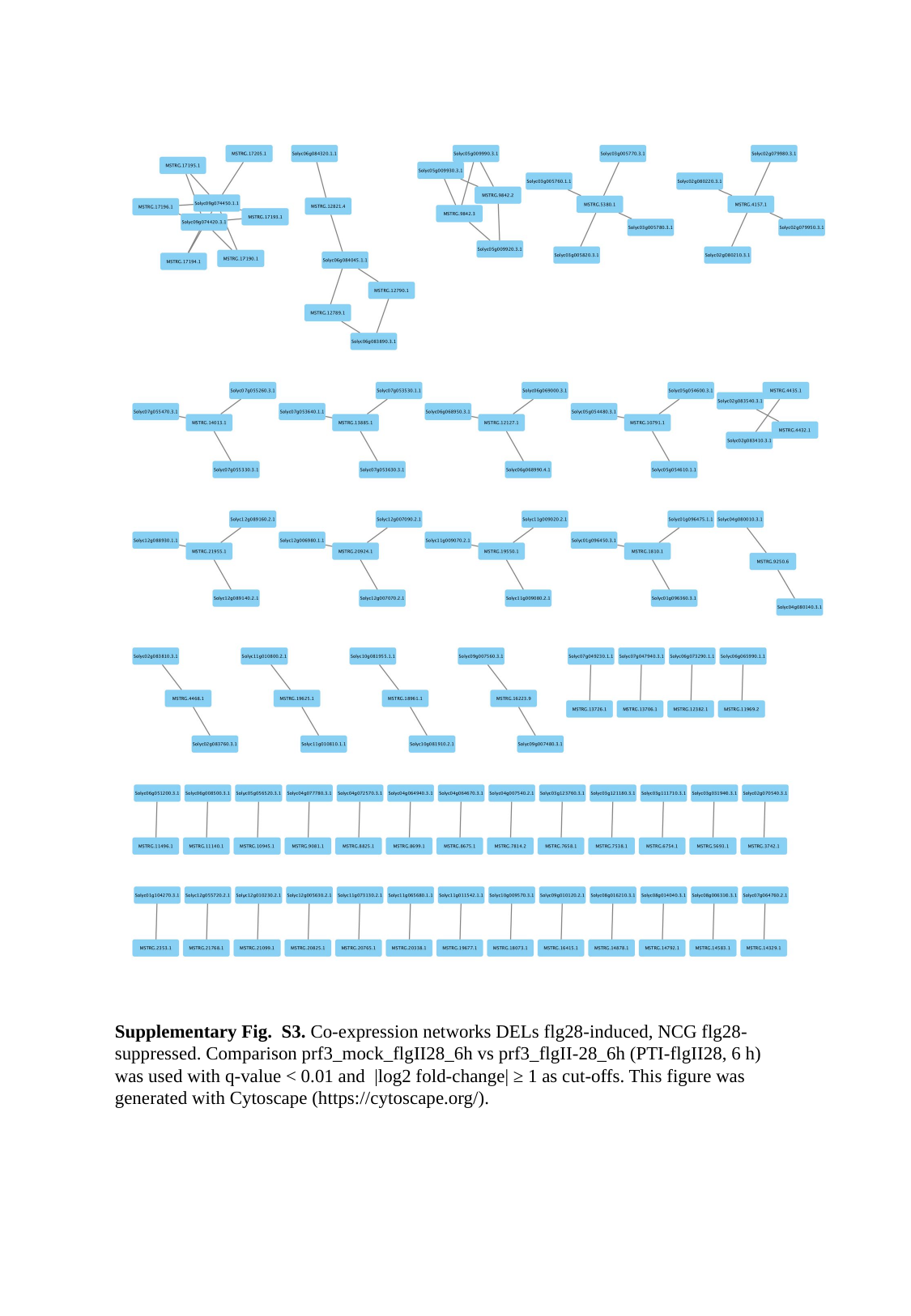

Supplementary Fig. S3. Co-expression networks DELs flg28-induced, NCG flg28-suppressed. Comparison prf3_mock_flgII28_6h vs prf3_flgII-28_6h (PTI-flgII28, 6 h) was used with q-value < 0.01 and |log2 fold-change| ≥ 1 as cut-offs. This figure was generated with Cytoscape (https://cytoscape.org/).

## Slide 4
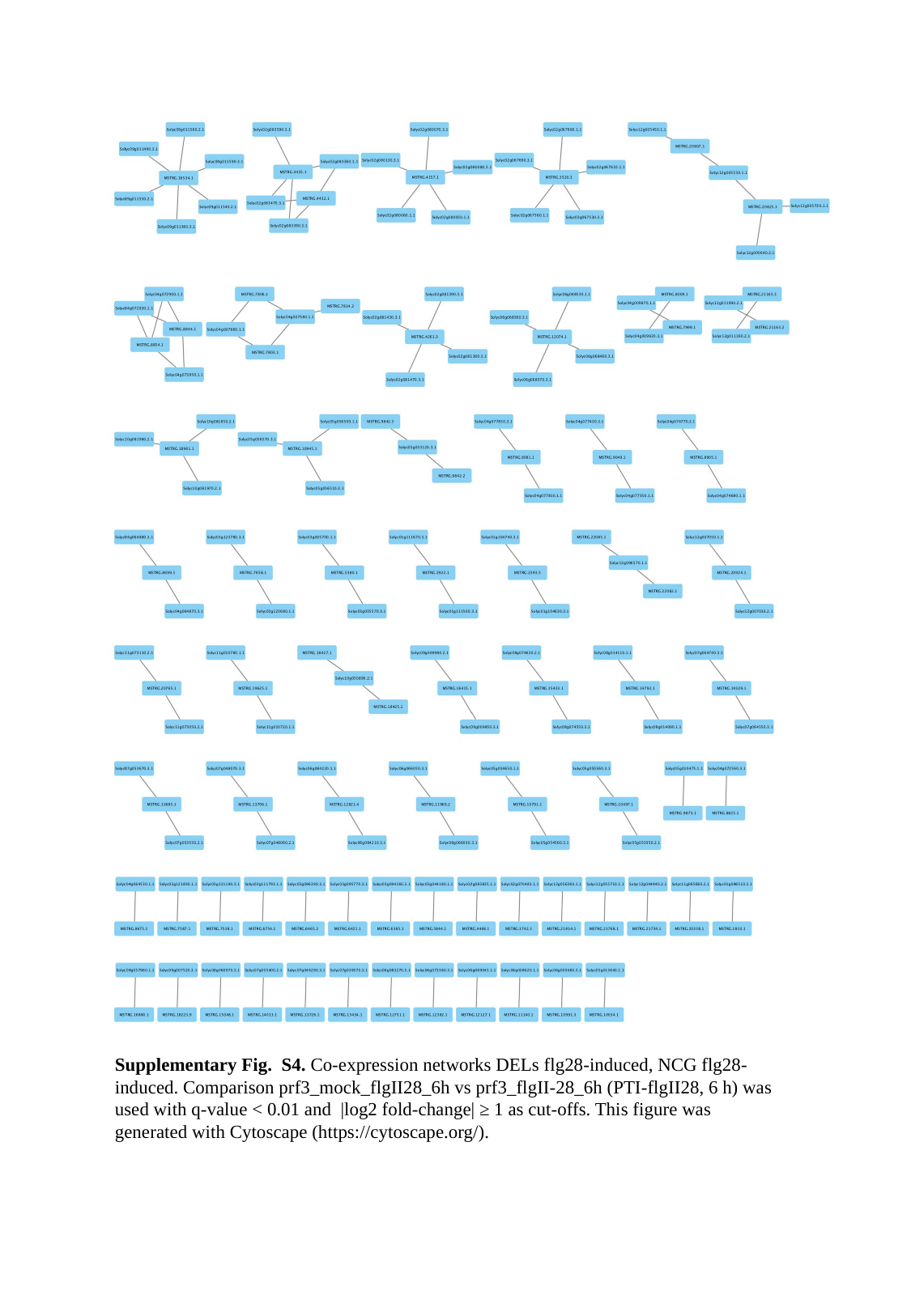

Supplementary Fig. S4. Co-expression networks DELs flg28-induced, NCG flg28-induced. Comparison prf3_mock_flgII28_6h vs prf3_flgII-28_6h (PTI-flgII28, 6 h) was used with q-value < 0.01 and |log2 fold-change| ≥ 1 as cut-offs. This figure was generated with Cytoscape (https://cytoscape.org/).

## Slide 5
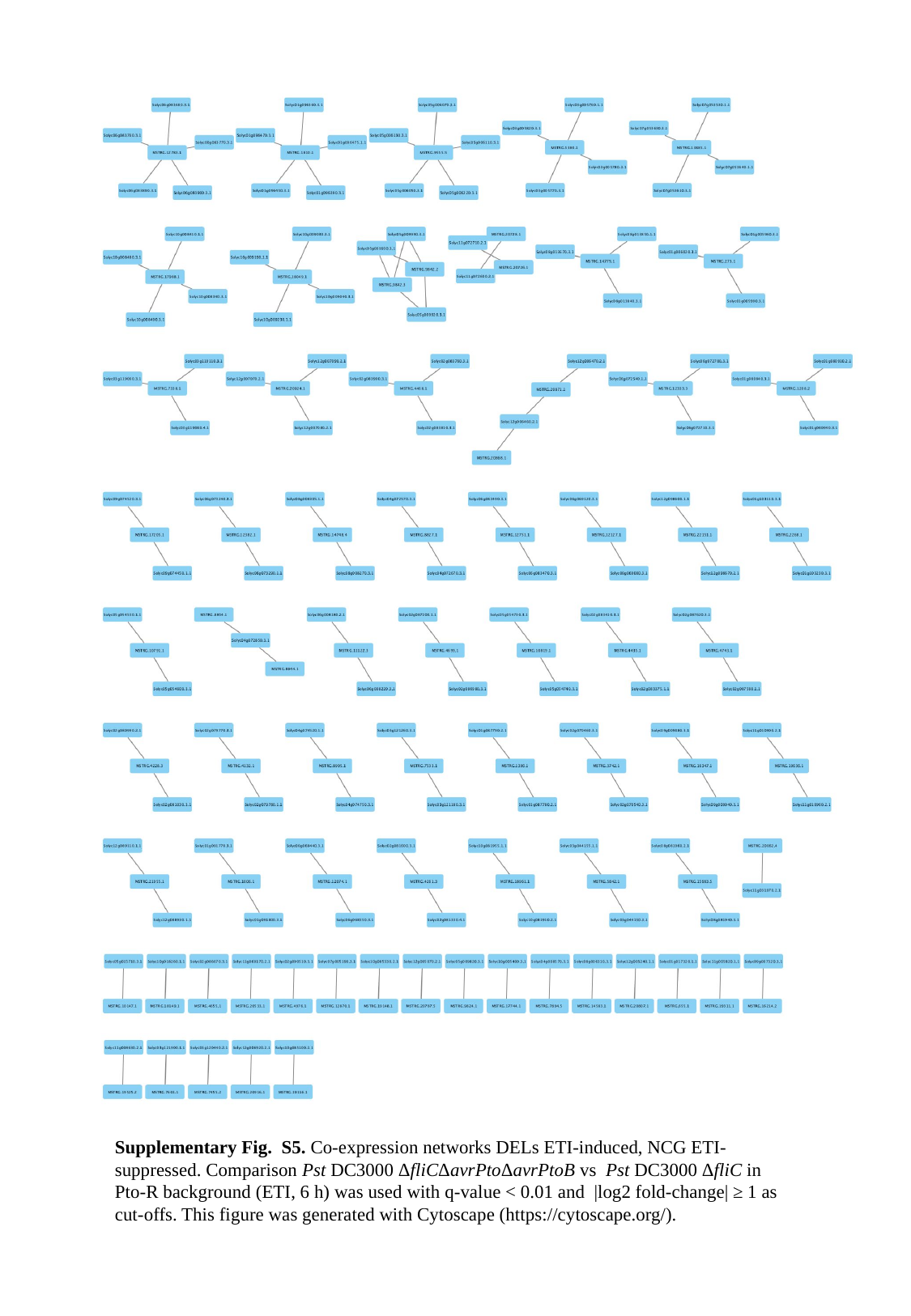

Supplementary Fig. S5. Co-expression networks DELs ETI-induced, NCG ETI-suppressed. Comparison Pst DC3000 ΔfliCΔavrPtoΔavrPtoB vs Pst DC3000 ΔfliC in Pto-R background (ETI, 6 h) was used with q-value < 0.01 and |log2 fold-change| ≥ 1 as cut-offs. This figure was generated with Cytoscape (https://cytoscape.org/).

## Slide 6
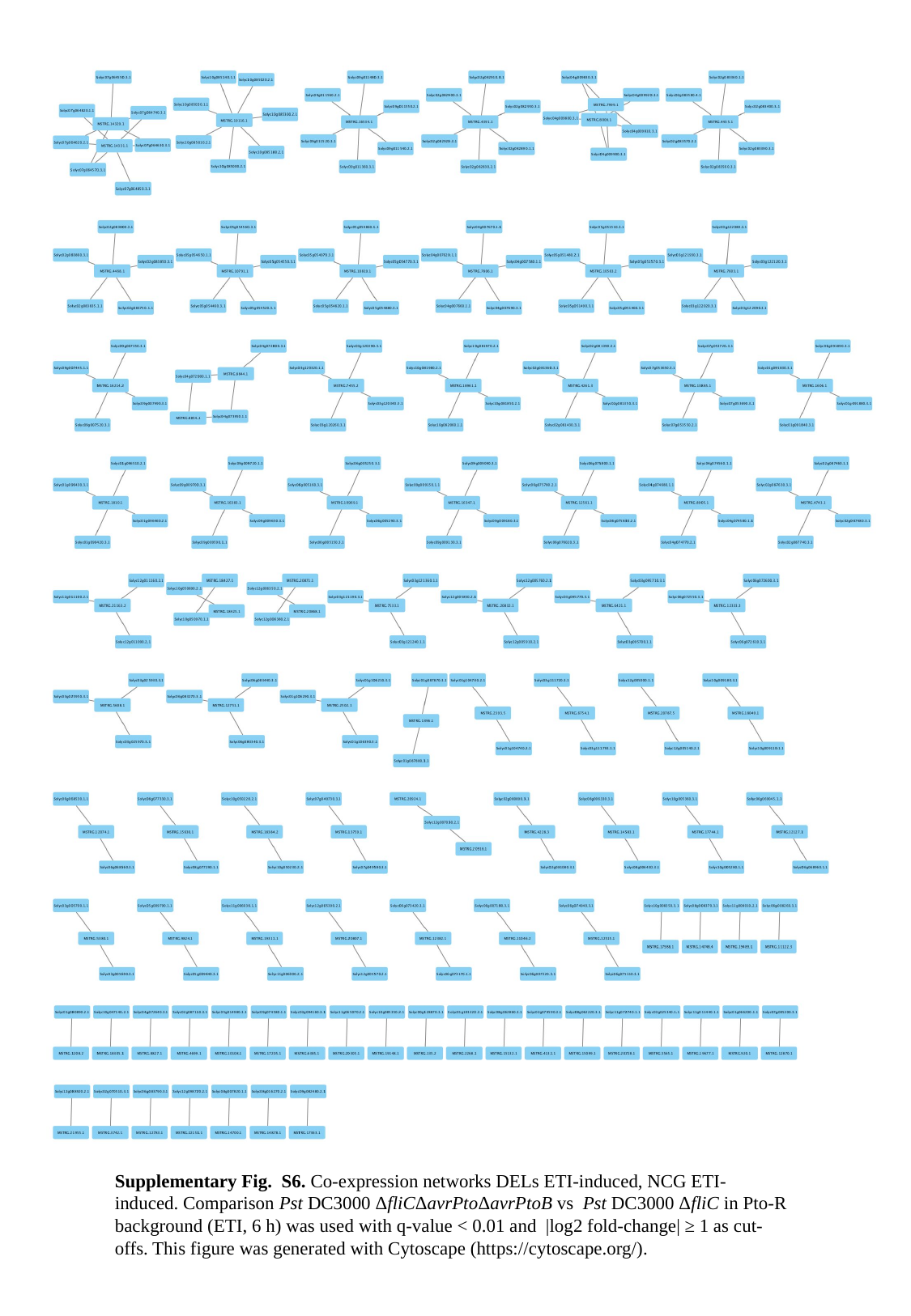

Supplementary Fig. S6. Co-expression networks DELs ETI-induced, NCG ETI-induced. Comparison Pst DC3000 ΔfliCΔavrPtoΔavrPtoB vs Pst DC3000 ΔfliC in Pto-R background (ETI, 6 h) was used with q-value < 0.01 and |log2 fold-change| ≥ 1 as cut-offs. This figure was generated with Cytoscape (https://cytoscape.org/).
