# Supplementary material for: Genome-wide analysis uncovers tomato leaf lncRNAs transcriptionally active upon Pseudomonas syringae pv. tomato challenge
Source: Sci Rep. 2021 Dec 31;11:24523. doi: 10.1038/s41598-021-04005-0 (PMC8720101; doi:10.1038/s41598-021-04005-0)
Supplement: Supplementary file 8 — Supplementary Table S7. [file 41598_2021_4005_MOESM8_ESM.pptx]

## Slide 1
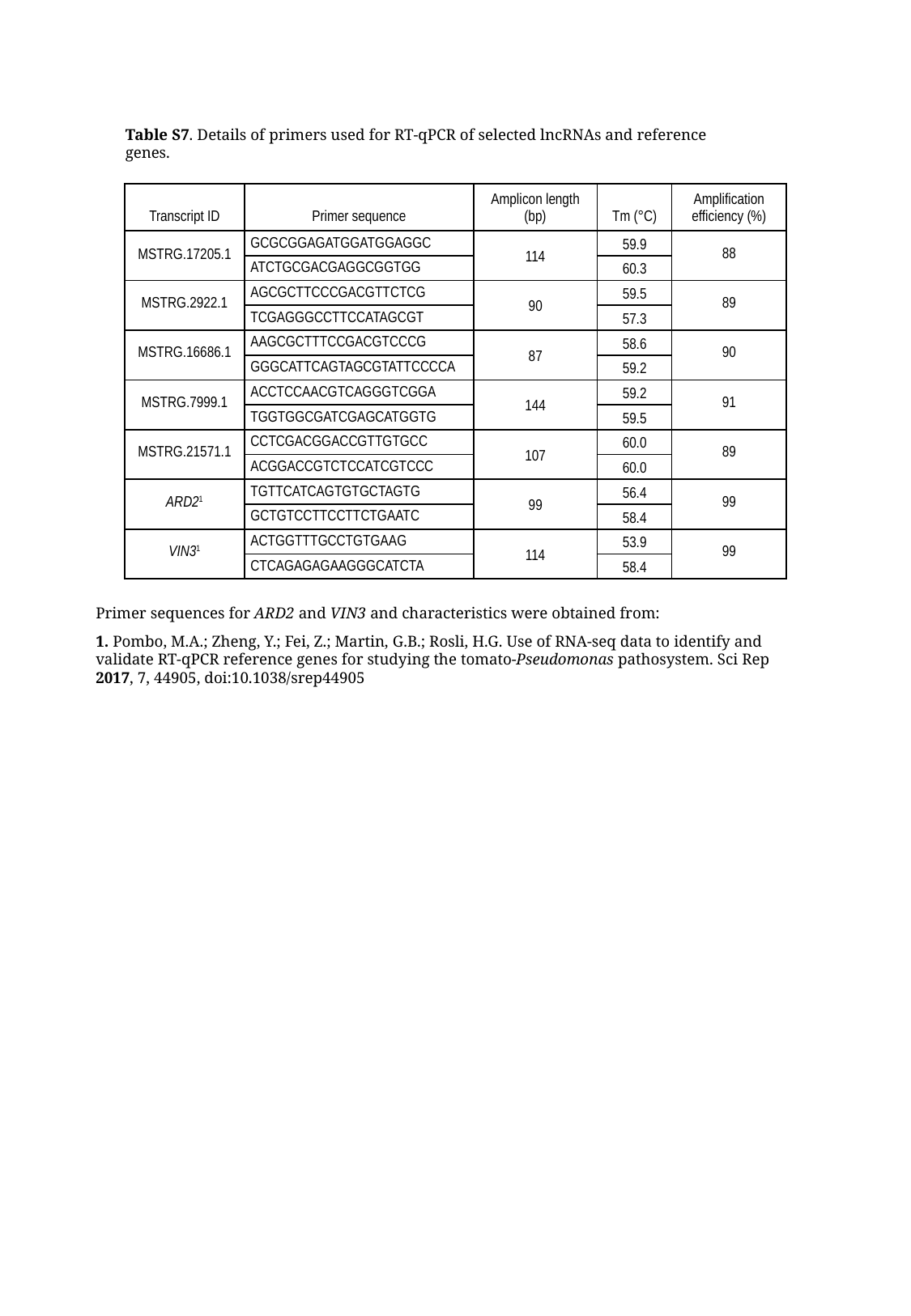

Table S7. Details of primers used for RT-qPCR of selected lncRNAs and reference genes.
| Transcript ID | Primer sequence | Amplicon length (bp) | Tm (°C) | Amplification efficiency (%) |
| --- | --- | --- | --- | --- |
| MSTRG.17205.1 | GCGCGGAGATGGATGGAGGC | 114 | 59.9 | 88 |
| | ATCTGCGACGAGGCGGTGG | | 60.3 | |
| MSTRG.2922.1 | AGCGCTTCCCGACGTTCTCG | 90 | 59.5 | 89 |
| | TCGAGGGCCTTCCATAGCGT | | 57.3 | |
| MSTRG.16686.1 | AAGCGCTTTCCGACGTCCCG | 87 | 58.6 | 90 |
| | GGGCATTCAGTAGCGTATTCCCCA | | 59.2 | |
| MSTRG.7999.1 | ACCTCCAACGTCAGGGTCGGA | 144 | 59.2 | 91 |
| | TGGTGGCGATCGAGCATGGTG | | 59.5 | |
| MSTRG.21571.1 | CCTCGACGGACCGTTGTGCC | 107 | 60.0 | 89 |
| | ACGGACCGTCTCCATCGTCCC | | 60.0 | |
| ARD21 | TGTTCATCAGTGTGCTAGTG | 99 | 56.4 | 99 |
| | GCTGTCCTTCCTTCTGAATC | | 58.4 | |
| VIN31 | ACTGGTTTGCCTGTGAAG | 114 | 53.9 | 99 |
| | CTCAGAGAGAAGGGCATCTA | | 58.4 | |
Primer sequences for ARD2 and VIN3 and characteristics were obtained from:
1. Pombo, M.A.; Zheng, Y.; Fei, Z.; Martin, G.B.; Rosli, H.G. Use of RNA-seq data to identify and validate RT-qPCR reference genes for studying the tomato-Pseudomonas pathosystem. Sci Rep 2017, 7, 44905, doi:10.1038/srep44905
